# Data on empirical investigation of direct and indirect effect of personality traits on entrepreneurs’ commitment of SMEs

**DOI:** 10.1016/j.dib.2018.05.097

**Published:** 2018-05-24

**Authors:** Ayoade Omisade Ezekiel, Ogunnaike Olaleke, Adegbuyi Omotayo, Fatai Lawal, Onakoya Femi

**Affiliations:** Covenant University, Nigeria

## Abstract

This data article presented the effect of the Big Five Personality traits on entrepreneurs’ commitment. 400 copies of questionnaire were administered to practicing entrepreneurs whom were members of a business guild in their annual end of year meeting and award day. 369 copies were duly filled and returned for use. Using statistic package for social science (SPSS 20) and Amos 22, correlation and regression analysis were used to find out the relationship between the two constructs and the strength of the relationship respectively. The Amos path diagram revealed the standardized estimates of the regression coefficient.

**Specifications Table**

**Table:** 

Subject area	*Entrepreneurship and Psychology*
More specific subject area	*Personality Traits and Entrepreneurs’ Commitment towards Business Performance*
Type of data	*SPSS data, figure and table*
How data was acquired	*Questionnaire survey*
Data format	*Raw, analysed, descriptive and statistical data.*
Experimental factors	*Sample consisted of SME entrepreneurs who were members of a business guild in South West Nigeria. Questionnaire was formulated around the Five Factors Model of Personality Traits – Agreeableness, Extraversion, Conscientiousness, Openness to Experience & Neuroticism- and Meyer and Allen (1997)*[1]*three components model of commitment – Affective, Continuous & Normative.*
Experimental features	*The unique trait in individual entrepreneur affect commitment towards business performance and Structural Equation Model (SEM) was used to validate the SPSS findings.*
Data source location	*South West Nigeria*
Data accessibility	*Data is included in this article*

**Value of the data**

• This data present a robust analytical and statistical technique to establish the interconnectedness that exists between entrepreneurs’ personality traits and commitment towards business performance.
• The data describe the demographic structure of the entrepreneurs which can help in government formulation of non-discriminative policy towards gender, age and level of education.
• The data contributes to the body of literature on entrepreneurs’ personality traits and commitment towards business performance.
• The data will aid academy discourse on the role of personality traits on the commitment of the entrepreneur.
• Our data can be compared with others collected from another part of the country or other part of the world.

## 1 Data

**Fig. 1:**
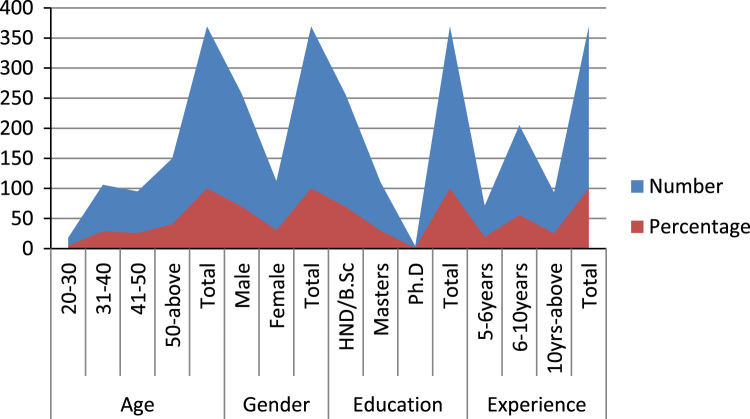
Demographic characteristics of respondents.

**Fig. 2:**
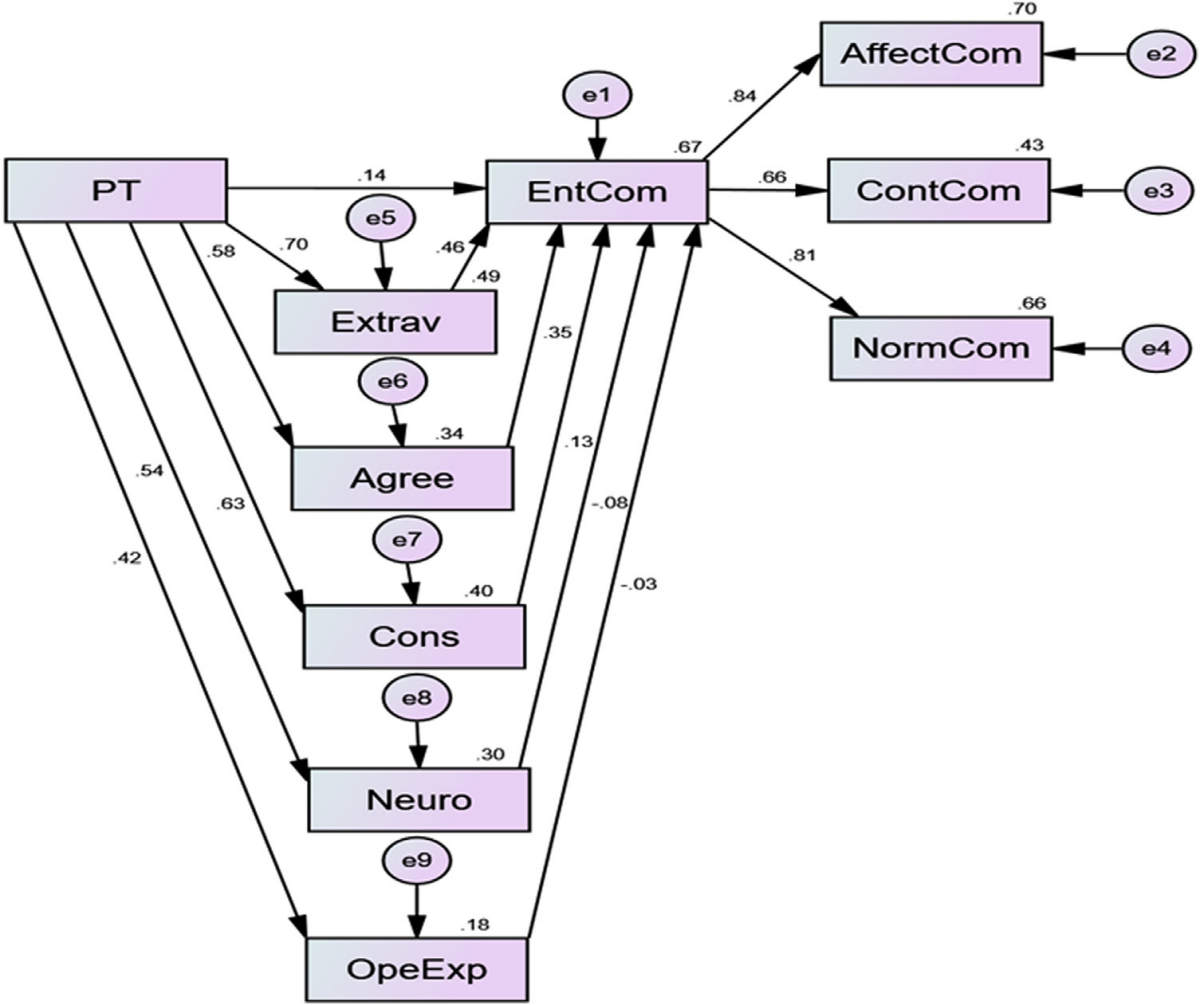
Modeling and interconnectedness estimation of personality traits and entrepreneurs׳ commitment. Notes: PT= Personality Traits, EntCom= Entrepreneurs’ Commitment, Extrav= Extraversion, Agree= Agreeableness, Cons= Conscientiousness, Neuro= Neuroticism, OpeExp= Openness to Experience, AffectCom= Affective Commitment, ContCom = Continuous Commitment, NormCom= Normative Commitment.

The data for this article emanated from 400 copies of questionnaire administered to SMEs owners in six states of south west Nigeria. Each of the state received percentage of its total registered member over all the total registered members in South West Nigeria. 369 copies which were 92.2% of number administered were returned and satisfied useful for analysis. Fig. 1 and Table 1 show the demographic nature of the respondents and the coded data was imputed into SPSS which is attached with this article. Structural Equation Modelling (SEM) was used to test the relationship between the independent variable (Personality traits) and the dependent variable (entrepreneurs’ commitment) [2], [3]. The standardised regression estimates is given in Fig. 2 and Table 2 below and the correlation coefficient is presented in Table 3.

**Table 1:** Demographic profile table.

**Demographics**	**Description**	**Number**	**Percentage**
**Age**	20–30	18	4.9
	31–40	106	28.6
	41–50	95	25.8
	50-above	150	40.7
	**Total**	**369**	**100.0**
**Gender**	Male	257	69.7
	Female	112	30.3
	**Total**	**369**	**100.0**
**Education**	HND/B.Sc	255	69.2
	Masters	110	29.7
	Ph.D	4	1.1
	**Total**	**369**	**100.0**
**Experience**	1–5	71	19.3
	6–10	205	55.6
	10-above	93	25.1
	**Total**	**369**	**100.0**

**Table 2:** Regression weights.

			Standardized estimate	Unstandardized estimate	S.E.	C.R.	P	Label
OpeExp	<---	PT	0.419	0.702	0.079	8.853	***	par_6
Extrav	<---	PT	0.703	1.14	0.06	18.971	***	par_11
Agree	<---	PT	0.583	0.649	0.047	13.782	***	par_12
Cons	<---	PT	0.63	1.092	0.07	15.558	***	par_13
Neuro	<---	PT	0.544	1.417	0.114	12.438	***	par_14
EntCom	<---	PT	0.139	0.178	par_2			
EntCom	<---	Agree	0.349	0.4	par_3			
EntCom	<---	OpeExp	−0.026	−0.02	par_7			
EntCom	<---	Neuro	−0.08	−0.039	par_8			
EntCom	<---	Extrav	0.455	0.359	par_9			
EntCom	<---	Cons	0.126	0.093	par_10			
ContCom	<---	EntCom	0.656	0.824	0.047	17.639	***	par_1
AffectCom	<---	EntCom	0.836	0.898	0.029	30.878	***	par_4
NormCom	<---	EntCom	0.812	1.279	0.045	28.202	***	par_5

**Table 3:** Correlations.

	PT	Cons	Extrav	Neuro		OpeExp	Agree	Ent Com	Norm Com	Affect Com	Cont Com
PT	1.000										
Cons	0.630	1.000									
Extrav	0.703	0.443	1.000								
Neuro	0.544	0.343	0.383	1.000							
OpeExp	0.419	0.264	0.295	0.228		1.000					
Agree	0.583	0.368	0.410	0.317		0.244	1.000				
EntCom	0.688	0.509	0.714	0.318		0.267	0.631	1.000			
NormCom	0.558	0.414	0.580	0.258		0.217	0.513	0.812	1.000		
AffectCom	0.575	0.426	0.597	0.265		0.223	0.528	0.836	0.679	1.000	
ContCom	0.451	0.334	0.468	0.208		0.175	0.414	0.656	0.533	0.548	1.000

## 2 Experimental design, materials and methods

Multi- stage sampling technique was employed and it was made up of;

1) Cluster Sampling as it included all the six states branches in south west Nigeria
2) Purposive sampling as only registered member were used, and
3) Convenience Sampling Technique as registered members that attended the monthly meeting.

The questionnaire was the instrument used to gather this data. There were 32 items in the questionnaire 8 statements on the bio-data, 9 statements on entrepreneur commitment and its factors [4], and 15 statements on Personality traits [5]. The questionnaire contains 5 point Likert items which ranges from strongly agree = 5, agree = 4, undecided = 3, disagree = 2, and strongly disagree = 1 [6]. The respondents are to evaluate based on their own understanding of the question or statement. Statistic package for social science (SPSS 20) and Amos 22 were the analytical instruments. Correlation and regression analysis were used to find out the connection between the two constructs and the strength of the relationship respectively. The Amos path diagram revealed the standardized estimates of the regression coefficients. Table 4 below shows the reliability test of the items in the instrument that shows the consistency and the repeatability and stability [7].

**Table 4:** Reliability test.

**Variables names**	**Cronbach׳s alpha**	**Number of items**
Affective commitment	0.751	3
Continuous commitment	0.723	3
Normative commitment	0.891	3
Extraversion	0.729	3
Agreeableness	0.740	3
Conscientiousness	0.856	3
Neuroticism	0.722	3
Openness to experience	0.769	3
