# Supplementary material for: Data on empirical investigation of direct and indirect effect of personality traits on entrepreneurs’ commitment of SMEs
Source: Data Brief. 2018 May 24;19:1171–5. doi: 10.1016/j.dib.2018.05.097 (PMC6139600; doi:10.1016/j.dib.2018.05.097)
Supplement: Supplementary file 1 — Supplementary material [file mmc1.doc]

Ayoade Omisade Ezekiel

Covenant University,

Ota,

Ogun State,

Nigeria

13 April, 2018

The Editor,

Data In Brief,

Dear Sir,

**DECLARATION OF CONFLICT OF INTEREST**

I, Ayoade Omisade Ezekiel and my colleagues write to declare that there is no conflict of interest traceable to our data paper “**Empirical investigation of direct and indirect effect of personality traits on entrepreneurs’ commitment of SMEs”**

Yours faithfully,


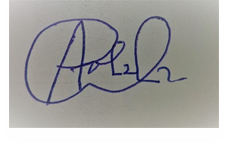


Ayoade Omisade Ezekiel (Corresponding Author)

+234-8037201846
